# Phenothiazines Modified with the Pyridine Ring as Promising Anticancer Agents

**DOI:** 10.3390/life11030206

**Published:** 2021-03-05

**Authors:** Beata Morak-Młodawska, Małgorzata Jeleń, Krystian Pluta

**Affiliations:** Department of Organic Chemistry, Faculty of Pharmaceutical Sciences, The Medical University of Silesia, Jagiellońska 4, 41-200 Sosnowiec, Poland; manowak@sum.edu.pl (M.J.); pluta@sum.edu.pl (K.P.)

**Keywords:** pirydobenzothiazines, dipyridothiazines, anticancer agents, gens analysis, apoptosis

## Abstract

Azaphenothiazines are the largest and most perspective group of modified phenothiazines, and they exhibit variety of biological activities. The review sums up the current knowledge on the anticancer activity of isomeric pyridobenzothiazines and dipyridothiazines, which are modified azaphenothiazines with one and two pyridine rings, respectively, against 10 types of cancer cell lines. Some 10-substituted dipyridothiazines and even 10-unsubstituted parent compounds, such as 10*H*-1,9-diazaphenothiazine and 10*H*-3,6-diazaphenothiazine, exhibited very potent action with the IC_50_ values less than 1 µg/mL and 1 µM against selected cancer cell lines. The strength of the anticancer action depends both on the tricyclic ring scaffolds and the substituents at the thiazine nitrogen atom. The review discusses the kind of the substituents, nature of tricyclic ring scaffolds with the location of the azine nitrogen atoms, the types of the cancer cell lines, and the mechanism of action.

## 1. Introduction

**Figure 1:**
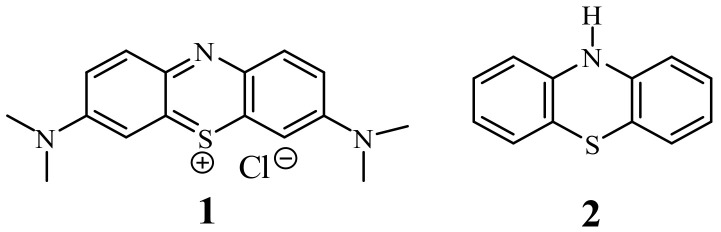
Methylene blue (**1**) and 10*H*-phenothiazine (**2**).

Phenothiazines are a very important class of heterocyclic compounds possessing a tricyclic dibenzo-[1,4]-thiazine ring system. This system, as the phenothiazinium salt, was for the first time synthesized by Caro in 1876 as a dye called methylene blue (**1**) [1]. Later in 1883, Bernthsen obtained free 10*H*-phenothiazine (**2**) in the reaction of sulfurization of diphenylamine with elemental sulfur [2] (Figure 1). In 1950s, the researchers at Rhône-Poulenc synthesized phenothiazines which exhibited antihistaminic and antipsychotic activities. Since this time, phenothiazines have been essential drugs (at least 100 phenothiazines) for the clinical treatment of psychotic disorders [3], among which chlorpromazine, thioridazine, fluphenazine, promethazine, and trifluoperazine are the most known. Those classical phenothiazines contained the dialkylaminoalkyl substituents at the thiazine nitrogen atom (in position 10) and additionally small substituents in position 2. They exhibit also analgesic, anti-emetic, antihelmentic, and antipuritic activities [3,4].

For a few decades, there has been an extremely high interest in searching for new derivatives of phenothiazines possessing different activities. The first approach is called drug repurposing or drug repositioning, and the use of known drugs for new biological targets decreases the cost of research, while maintaining quality and proven safety [5,6,7]. Phenothiazines with thioridazine at the lead are regarded as the most up and coming in a treatment of the multidrug resistant tuberculosis [8,9,10,11], cancer cells [5,6,7,12,13,14,15,16], and neurodegenerative diseases [13]. They also exhibit antiviral, anti-emetic, antipruritic, anti-inflammatory, antitussive, analgesic, antibacterial, and antifungal activities [13,14,17].

The second approach is based on the synthesis of new structurally modified phenothiazines. This modification involves the insertion of new substituents at the nitrogen atom, oxidation of the sulfide group into sulfoxide and sulfone groups in the thiazine ring, and the exchange of one or two benzene rings with other rings (homoaromatic and heteroaromatic). The last modification is the most perspective because it allows the formation of not only a new phenothiazine scaffold (not only tricyclic but also tetracyclic and pentacyclic), but also allows to introduce new substituents. The introduction of the azine ring such as pyridine, pyridazine, pyrimidine, pyrazine, or quinoline in the place of the benzene ring, leads to new derivatives of phenothiazines, azaphenothiazines. Over 50 types of azaphenothiazine scaffolds are known which belong to varied heterocyclic ring systems [18,19].

The anticancer activity of classical and modified phenothiazines was summarized for the first time by Motohashi in the book chapter in the late 1980s [20]. Later, he with his research groups started extensive studies on the anticancer activity of new phenothiazines which were the inspiration to other research groups. They synthesized modified tricyclic phenothiazines with amidoalkyl, sulfonamidoalkyl, and chloroethylureidoalkyl substituents in position 10 and replaced the benzene ring with naphthalene. These phenothiazines exhibited cytotoxic activities against ten different human tumor cell lines: leukemia, melanoma, small cell lung, colon, central nervous system, renal, breast, ovarian, and prostate tumors. Their results were published in over 50 original papers and reviews [21,22,23,24,25,26,27,28,29].

The modified phenothiazines exhibit not only anticancer activity, but a wide range of other activities such as the reversal of multidrug resistance, anti-inflammatory, antibacterial, antiviral, antiplasmid, cholinesterase inhibitory, antioxidant, and antihyperlipidemic and are considered potential agents in the treatment in Alzheimer’s and Creutzfeldt–Jakob diseases, published in hundreds of original papers and patents, and summarized in numerous comprehensive review papers and chapters [18,19,30,31,32,33,34,35,36,37,38]. 

The modification of the phenothiazine structures with the pyridine ring leads to different pyridobenzothiazines and dipyridothiazines, also named x-monoazaphenothiazines and x,y-diazaphenothiazines, the largest but also most diverse family of azaphenothiazines. The aim of this review is to present comprehensively anticancer activities of those azaphenothiazines.

## 2. Pyridobenzothiazines

Pyridobenzothiazines represent linearly condensed tricyclic ring systems where the 1,4-thiazine ring is fused with the benzene and pyridine rings along the C-C bonds. As they differ from classical phenothiazines in the presence of the additional nitrogen atom instead of the carbon atom, they are named azaphenothiazines (1-, 2-, 3- and 4-azaphenothiazines).

### 2.1. 1-Azaphenothiazines (Pyrido[3,2,-b]benzo[1,4]thiazines)

**Figure 2:**
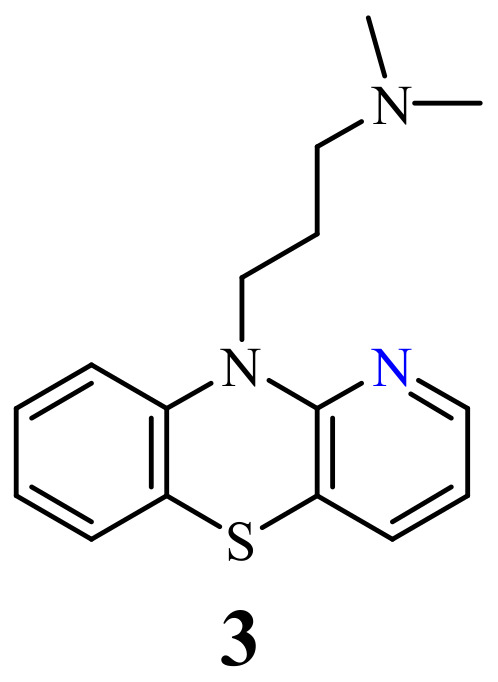
Prothipendyl (**3**).

**Figure 3:**
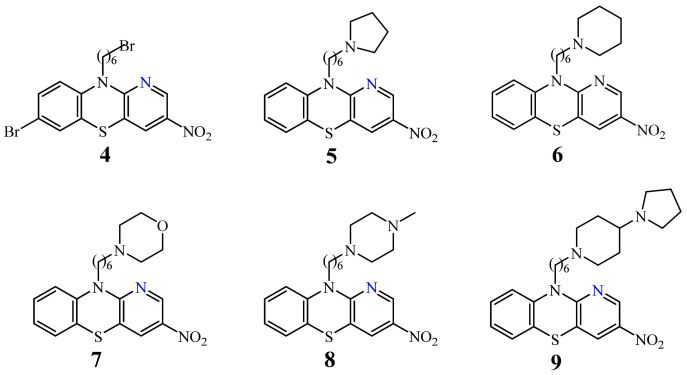
A family of the most acive10-aminoalkylated azaphenothiazines (**4**–**9**) with a hexyl linker.

Of the 1-azaphenothiazines, antitumor activity has so far been described only for 10-substituted derivatives including a dimethylaminopropyl group (**3**), called prothipendyl (Figure 2), and a hexyl chain containing amino and nitro groups or a bromine atom at the end (**4**–**9**) (Figure 3).

Prothipendyl, the most useful pyridobenzothiazine, was synthesized in 1960 [39] and is known under brand name Dominal and Tolnate; it is still in use in treatments of anxiety and agitation in psychotic syndromes [40,41,42,43,44].

The anticancer activity of prothipendyl was investigated recently using the cultured glioblastoma SNB-19, melanoma C-32, breast cancer MCF-7, and ductal carcinoma T47D cell lines with the normal human fibroblast (HFF-1) cell line used as a control. The cell lines used showed a different sensitivity to prothipendyl. The MCF-7 and the C32 cells lines were found to be the most sensitive with the IC_50_ value of 23.2 µg/mL and 28.1 µg/mL, respectively. The T47D and the SNB19 cells lines were the most resistant for the tested compound (with IC_50_ of 32.3 µg/mL and 36.6 µg/mL, respectively). Prothipendyl was found to be non-toxic (IC_50_ > 50 µg/mL) against the normal human fibroblast (HFF-1) cell line in comparison with cisplatin used as a reference (IC_50_ = 8.2 µg/mL) [45,46,47].

A set of twenty-one novel 10-substituted 1-azaphenothiazines (**4**–**9**) containing aminohexyl and bromohexyl groups together with a nitro group in the pyridine ring (Figure 3) were investigated for their action against six cancer cell lines (malignant brain cancer T98G, lung cancer H460, thyroid cancer SNU80, oral cancer KB, blood cancer THP1, and blood cancer U937) and two normal cell lines (the normal lung fibroblast MRC5 and the normal embryonic kidney cells HEK293) with the actinomycin D used as the standard drug in the studies.

Eleven of the compounds tested exhibited distinct inhibitory action against selected cancer lines (IC_50_ < 10 µg/mL) and were a few times more effective in comparison to a standard drug actinomycin D. Compound **6** (with the piperidinylhexyl group) showed the highest activity against the lung cancer H460, the malignant brain cancer T98G, and the thyroid cancer SNU80 with the IC_50_ values of 2.27–3.8 µg/mL, respectively. Similarly active were derivatives **9** (with the pyrrolidinyl-piperidinylhexyl group) against the H460 and SNU80 cell lines (IC_50_ = 2.1 and 2.3 µg/mL), **7** (morpholinylhexyl group), and **5** (pyrrolidinylhexyl) against H460 line (IC_50_ = 2.5 and 2.7 µg/mL). The derivative **4** was active against those three cell lines showing the IC_50_ values of 3.8–6.2 µg/mL [48].

Very recently, prothipendyl exhibited antiviral action against a mosquito-transmitting alphavirus (CHIKV) inducing CHIK fever [49,50].

Other derivatives of 1-azaphenothiazine exhibited mainly antihistaminic, anticholinergic, anti-emetic, antitussive, neuropharmacological, antibacterial, and antitubercular activities and were the subject of many publications and reviews [51,52,53,54,55,56,57,58,59,60,61,62].

### 2.2. 2-Azaphenothiazines (Pyrido[4,3-b]benzo[1,4]thiazines)

**Figure 4:**
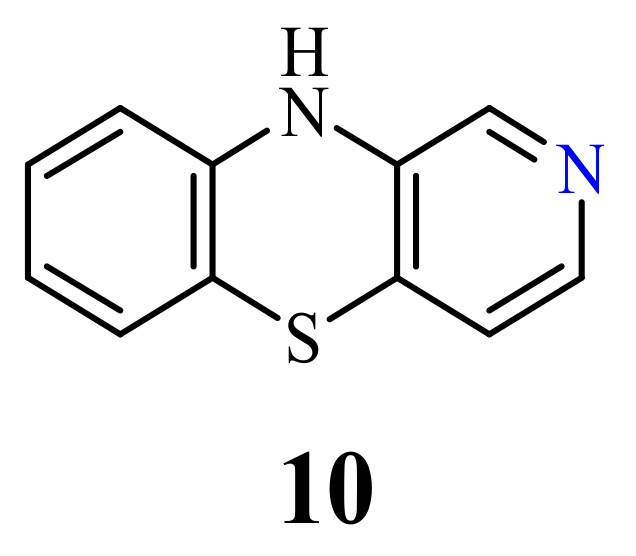
10*H*-2-azaphenothiazine (**10**).

Despite the fact that 10*H*-2-azaphenothiazines (**10**) (Figure 4) was synthesized in the late 1950s [63,64], there are no reports in the literature about the antitumor activity of molecules containing the 2-azaphenothiazine skeleton. Instead of that, the aminoalkyl derivatives exhibited antipsychotic and sedative properties [65].

### 2.3. 3-Azaphenothiazines (Pyrido[3,4-b]benzo[1,4]thiazines)

**Figure 5:**
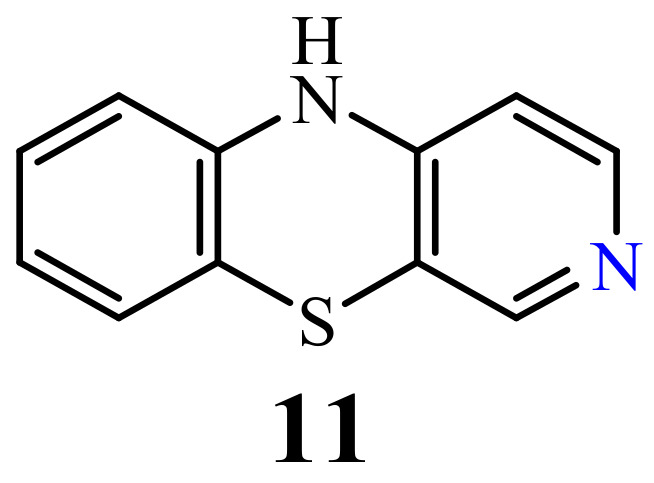
10*H*-3-azaphenothiazine (**11**).

Although the synthesis of 10*H*-3-azaphenothiazine (**11**) (Figure 5) was described in the 1960s [66], antitumor activity has not yet been described but its derivatives exhibited sedative, hypnotic, anticonvulsant, and hypotensive activity [65,66]

### 2.4. 4-Azaphenothiazines (Pyrido[2,3-b]benzo[1,4]thiazines)

**Figure 6:**
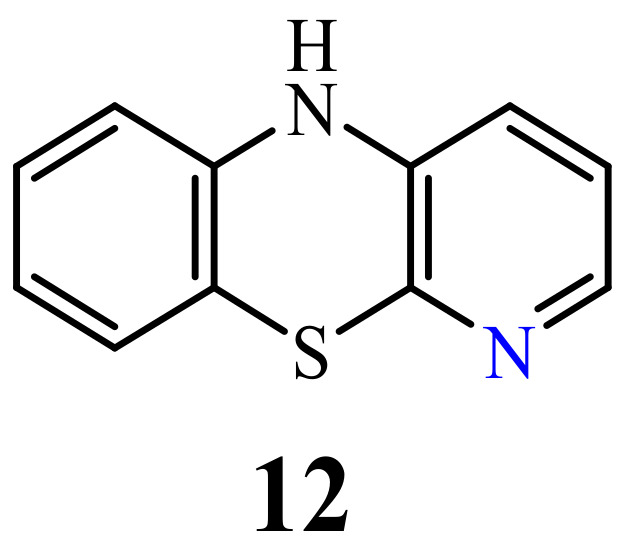
10*H*-4-Azaphenothiazine (**12**).

10*H*-4-Azaphenothiazine (**12**) (Figure 6) was obtained in 1958 [67]. Until now, only studies on their potential activity as allergy inhibitors were conducted for compounds containing the 4-azaphenothiazine skeleton [68].

As was discussed above, pyridobenzothiazines are not intensively explored nowadays, despite their wide range of biological activities.

The information provided on anticancer activity of pyridobenzothiazines was summarized in Table 1.

## 3. Dipyridothiazines

Dipyridothiazines mean linearly condensed tricyclic ring systems where the 1,4-thiazine ring is fused with two pyridine rings along the C-C bonds. As they contain two additional nitrogen atoms (instead of two carbon atoms) in comparison with classical phenothiazines, they are named diazaphenothiazines. Out of ten theoretically possible dipyridothiazine systems, only six have been known as 1,6-, 1,8-, 1,9-, 2,7-, 3,6-, and 3,7-diazaphenothiazines. This small amount of well-known dipyridothiazine types is a result of the difficulties in their synthesis involving orto disubstituted (2,3- and 3,4-)pyridines [19].

### 3.1. 1,6-Diazaphenothiazines (Dipyrido[2,3-b;2’,3’-e][1,4]thiazines)

**Figure 7:**
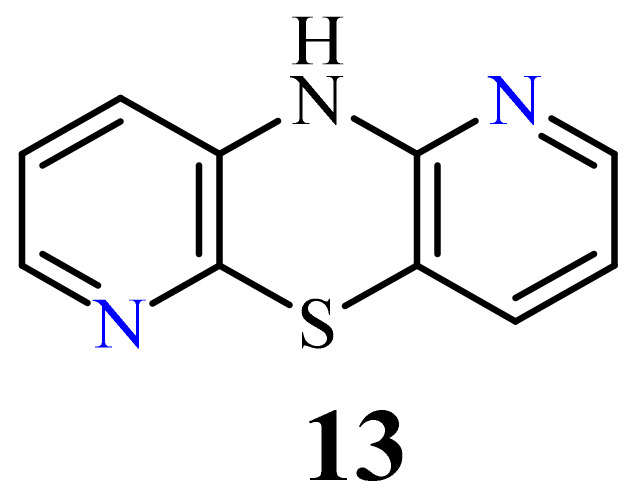
10*H*-1,6-Diazaphenothiazine (**13**).

10*H*-1,6-Diazaphenothiazine (**13**) (Figure 7) was prepared in 1958 and was converted into a dimethylaminopropyl derivative. This compound exhibited slight synergic effect with known biomolecules such as morphine and barbiturates and weak action of lowering body temperature in relation to chlorpromazine [76].

**Figure 8:**
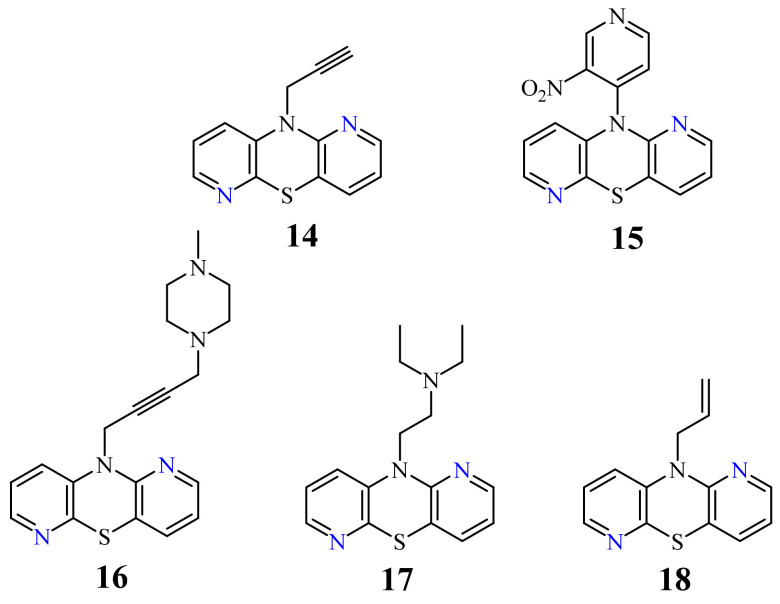
A family of anticancer 1,6-diazaphenothiazines with the alkyl, heteroaryl, dialkylaminoalkyl, dialkylaminoalkynyl, and alkenyl groups **14**–**18**.

A large family of 1,6-Diazaphenothiazine derivatives (Figure 8) with the alkyl, heteroaryl, amidoalkyl, and dialkylaminoalkynyl groups was synthesized in 2016 and tested for their anticancer action against the glioblastoma SNB-19, melanoma C-32, and breast cancer MCF-7 cell lines [47]. The compounds exhibited diverse levels of activity (IC_50_ in the range of 3.9 to 49.1 µg/mL) depending on the type of substituent and the type of cell line. The parent compound, 10*H*-1,6-diazaphenothiazine (**13**), and compounds **14** and **15** (containing the propargyl and nitropyridinyl substituents at the thiazine nitrogen atom) were found to be more active (IC_50_ = 4.8, 3.9 and 4.6 µg/mL) in relation to cisplatin as a reference compound (7.4 µg/mL) against the breast cancer MCF-7 cells. The derivative **16** (containing the methylpiperazinylbutynyl substituent) was as effective as cisplatin (7.5 µg/mL). 10*H*-1,6-Diazaphenothiazine (**13**) and compound **17** (containing the diethylaminoethyl substituent) were slightly more effective (7.5 and 6.6 µg/mL) than cisplatin (7.8 µg/mL) against the C-32 cells. The derivative **18** (with the allyl substituent) was the most effective against the SNB-19 cells with the IC_50_ values of 18.9 µg/mL. The most anticancer active compounds were nontoxic with reference to normal fibroblasts HFF-1 with the IC_50_ values exceeded 50 µg/mL [47].

Further studies looking for the structure–activity relationship (SAR) related to lipophilicity analysis were performed; however, they did not show the absolute dependence of the anticancer activity on the lipophilic properties [77].

**Figure 9:**
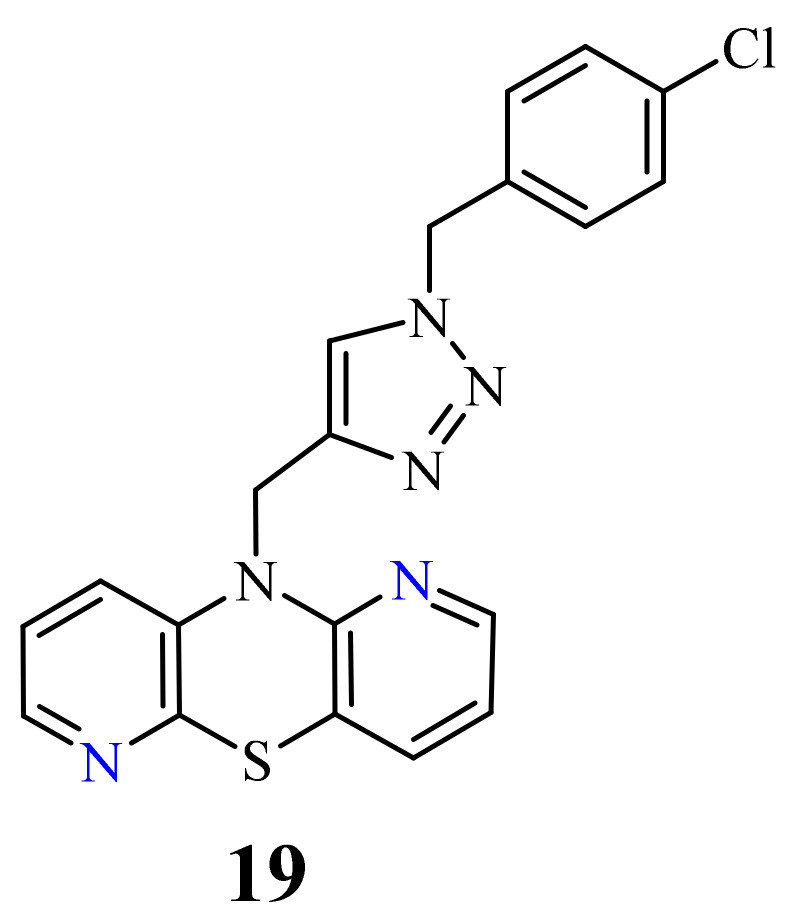
1,6-Diazaphenothiazine with the 1,2,3-triazole ring and *p*-chlorobenzyl substituent (**19**).

Recently, novel 1,2,3-triazole-dipyridothiazine hybrids containing the 1,6-diazaphenothiazine system and selected *p*-substituted benzyl substituents at the 1,2,3-triazole ring were obtained. The compound **19** (Figure 9) with the *p*-chlorobenzyl substituent showed an extremely promising anticancer activity (IC_50_ = 0.25–4.66 µM) in relation to the glioblastoma SNB-19, the colorectal carcinoma Caco-2, the lung cancer A549, and the breast cancer MDA-MB231 [69].

The compound was nontoxic against the normal fibroblasts NHDF with IC_50_ > 100 µM. This derivative was selected to study the mechanism of anticancer activity using RT-qPCR method. The influence on the gene transcriptional activities of proliferation marker (*H3*), the cell cycle regulator (*TP53* and *CDKNIA*), and the intracellular apoptosis pathway (*BCL-2* and *BAX*) were analyzed. The compound greatly affected the mRNA copy number of histone *H3* gene in all cancer lines, which had an influence on a modification of the chromatin structure in the cells and significantly interfered with the amount of mRNA copies of the *TP53* in all investigated cancer lines. There was also a strong decrease in the *CDKN1A* copies in all cancer cells, which can suggest a possibility of participation in cell cycle arrest. The derivative reduced remarkably the expression of the *BCL-2* in SNB-19, A549, and MDA-MB231 cancer lines, but in the Caco-2 cell line there was a slight increase. The analysis of expression of *H3*, *TP53*, *CDKN1A*, *BCL-2*, and *BAX* genes revealed that this compound inhibited the proliferation in all cells and activated mitochondrial events of apoptosis [69].

### 3.2. 1,8-Diazaphenothiazines (Dipyrido[2,3-b;3’,4’-e][1,4]thiazines)

**Figure 10:**
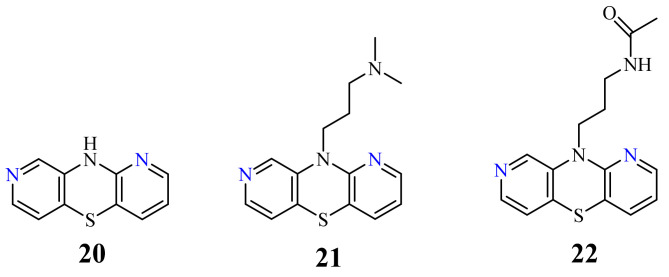
Biologically active 1,8-diazaphenothiazines **20**–**22**.

A group of 1,8-diazaphenothiazines was first reported in 2015. The biological potential of the compounds was the subject of both the publication and the patent [70,71]. 10*H*-1,8-Diazaphenothiazine **20** (Figure 10) and its 10-substituted derivatives containing various groups, such as the alkyl, heteroaryl, dialkylaminoalkyl, amidoalkyl, and sulfonamidoalkyl, were tested for their pharmacological activities. The test of proliferative response of the human peripheral blood mononuclear cells (PBMC) induced by the phytohemagglutinin A (PHA) exhibited a meaningful action of compound **20** and the derivative **21** (containing the dimethylaminopropyl substituent, Figure 10) at concentration of 50 µg/mL. These compounds showed the strongest inhibition of the tumor necrosis alpha (TNF-α) production generated by the lipopolysaccharide (LPS). All compounds revealed slight cytotoxic properties with the inhibition of PBMC. The most promising derivatives **20**–**22** (**22** containing the acetamidopropyl substituent) were screened against the leukemia L-1220 and the colon carcinoma SW-948 cell lines. The most effective compound turned out the parent compound **20**, exhibiting similar effect as cisplatin against the cancer cells of carcinoma SW-948 and leukemia L-1210 at 5 and 10 µg/mL, respectively.

**Figure 11:**
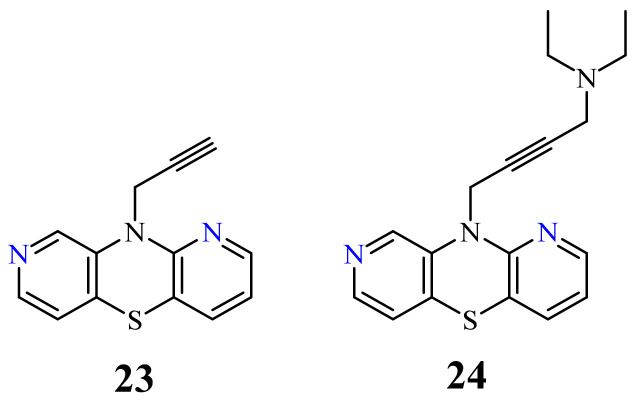
10-Propargyl-1,8-diazaphenothiazine (**23**) and its derivative **24**.

Another group of 1,8-diazaphenothiazines was obtained by a transformation of the 10-propargyl-1,8-diazaphenothiazine (**23**) (Figure 11) into the dialkylaminobutynyl derivatives. Its anticancer activity was evaluated against the cell lines of human glioblastoma SNB-19, melanoma C-32, and breast cancer T-47D using cisplatin as a reference compound. The compounds were characterized by a moderate activity (IC_50_ in the range of 26 to 46 µg/mL) which was dependent on both the type of substituent and the type of cell line. The most active compound was 10-diethylaminobutynyl-1,8-diazaphenothiazine (**24**) (IC_50_ = 26.1 µg/mL) against the melanoma C-32 [45].

In order to search for quantitative relationships between physicochemical and pharmacological properties of the investigated compounds, preliminary QSAR (Quantitative Structure-Activity Relationship) studies were undertaken for anticancer and immunosuppressant 1,8-diazaphenothiazines. For this group of diazaphenothiazines, some correlations between the TNF-α inhibition, anticancer activity, and lipophilicity were observed [78].

**Figure 12:**
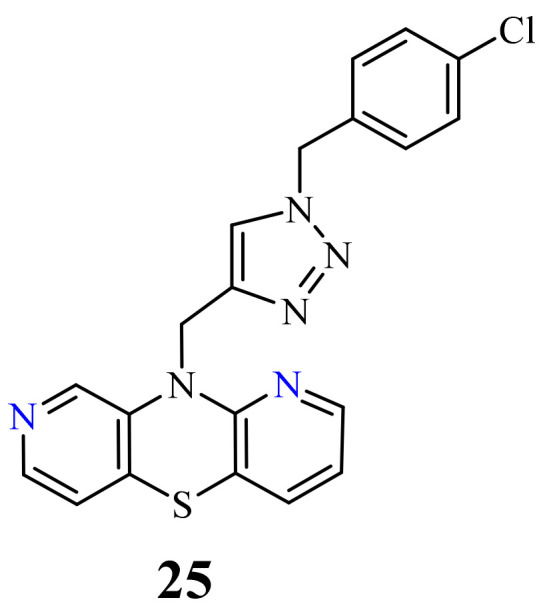
The hybrids of 1,8-diazaphenothiazine and 1,2,3-triazole **25** with a strong anticancer activity against A549.

A group of novel hybrids of the 1,8-diazaphenothiazine and 1,2,3-triazole systems were obtained and tested for their cytotoxicity against the glioblastoma SNB-19, colorectal carcinoma Caco-2, lung cancer A549, and breast cancer MDA-MB231 cell lines. The anticancer activity of this group of derivatives was differential (IC_50_ = 1.8–49 µM). The compound **25** (Figure 12) with the *p*-chlorobenzyl substituent showed a strong promising anticancer activity (IC_50_ = 1.82 µM) in relation to the lung cancer A549. However, this activity was lower than that of the isomeric 1,6-diazaphenothiazine **19** (IC_50_ = 0.25 µM) [69].

### 3.3. 1,9-Diazaphenothiazines (Dipyrido[2,3-b;2’,3’-e][1,4]thiazines).

**Figure 13:**
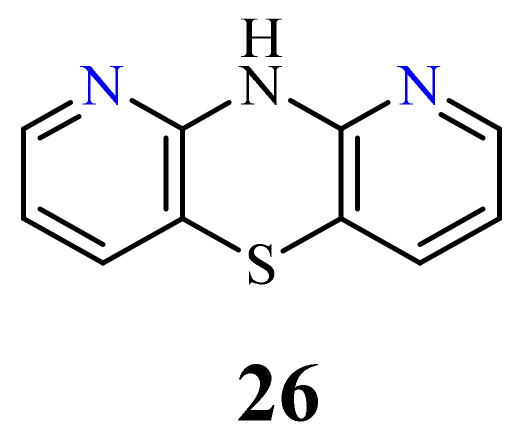
10*H*-1,9-Diazaphenothiazines (**26**).

The structure of 10*H*-1,9-diazaphenothiazine (**26**) (Figure 13) was first described in a US patent in 1957. However, the information contained in this report was very cursory and did not present the biological aspects of this compound [79].

**Figure 14:**
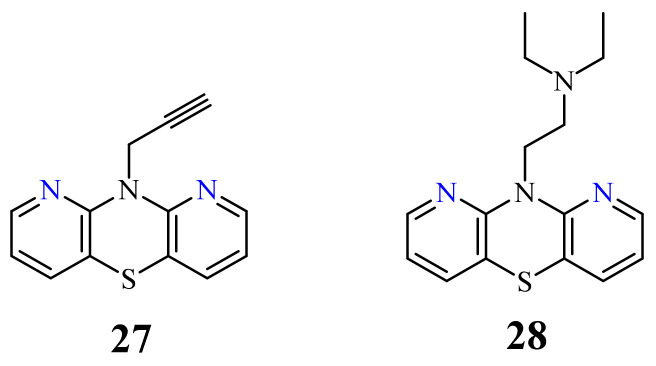
The most anticancer active 10-propynyl- (**27**) and 10-dietylaminoetyl-1,9-diazaphenothiazine (**28**).

Very recently, an efficient synthesis of 10*H*-1,9-diazaphenothiazine (**26**) was described, along with a complete structural analysis, as well as new 10-substituted derivatives containing alkyl, alkynyl, and dialkylaminoalkyl substituents. These compounds with various substituents were screened for their anticancer activity against using the glioblastoma SNB-19, melanoma C-32, and breast cancer MDA-MB-231 cell lines [72]. The parent compound 10*H*-1,9-diazaphenothiazine (**26**) was very active against melanoma C-32 (IC_50_ = 3.83 µM), even more potent than cisplatin (IC_50_ = 13.2 µM), but inactive against other lines. These derivatives induced a varying anticancer activity. The most promising derivatives in this group were compounds **27** and **28** with the propynyl and diethylaminoethyl groups (Figure 14).

10-Propynyl-1,9-diazaphenothiazine (**27**) was highly active against the glioblastoma SNB-19 (IC_50_ = 3.85 µM) and the melanoma C-32 (IC_50_ = 3.37 µM). 10-Diethylaminoethyl-1,9-diazaphenothiazine (**28**) was even more active against the glioblastoma SNB-19 with an IC_50_ value of 0.34 µM, and the breast cancer MDA-MB-231 cell line with IC_50_ = 2.13 µM. For those two compounds, the expression of *H3*, *TP53*, *CDKN1A*, *BCL-2*, and *BAX* genes was detected by the RT-qPCR method. The analysis of the gene expression revealed that both compounds selectively reduced the expression of *H3* and *TP53*, and enhanced the expression of *CDKN1A* in the examined cell lines. The gene expression ratio of *BAX/BCL-2* indicated the induction of mitochondrial apoptosis in two cancer cell lines (SNB-19 and MDA-MB-231). In the melanoma C-32 cell line, the transcriptional gene activity suggests a different way of cell death. The proteome profiling study showed the most probable compound action on SNB-19 cells through the intrinsic mitochondrial pathway of apoptosis [72].

### 3.4. 2,7-Diazaphenothiazines (Dipyrido[3,4-b;3’,4’-e][1,4]thiazines)

A family of 2,7-diazaphenothiazines was widely described in the literature both due to good chemical properties and highly significant bioactive properties. The first reports on the synthesis of this new heterocyclic system appeared in the literature at the beginning of the 21st century [80,81].

**Figure 15:**
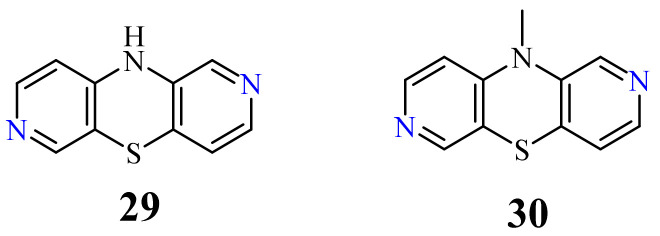
Bioactive 2,7-diazaphenothiazines **29** and **30**.

The parent compound 10*H*-2,7-diazaphenothiazine (**29**) and its methyl derivative **30** (Figure 15) strongly reduced the proliferative response of PBMC to PHA at 10 µg/mL. The former derivative exhibited very suppressive effect (72% at 1 µg/mL) in relation to the secondary humoral response to sheep red blood cells (SRBC). In addition, this derivative importantly reduced the delayed-type hypersensitivity (DTH) response to ovalbumin in the in vivo test using mice. In the experiment of cytokine production induced by LPS, this compound totally inhibited the IL-6 production at 100 µg/mL and moderately inhibited the TNF-α production at 10 µg/mL. The group of tested 2,7-diazaphenothiazines had low toxicity toward mouse splenocytes [82].

10*H*-2,7-diazaphenothiazine (**29**), and its selected 10-substituted derivatives containing various substituents, were also screened for their anticancer activity in the National Cancer Institute, Bethesda, MD, USA. In a preliminary experiment, the most promising compound turned out to be parent compound **29**, which was screened on about 60 cell lines including leukemia, melanoma, non-small cell cancer, colon cancer, CNS cancer, ovarian cancer, renal cancer, prostate cancer, and breast cancer. The strongest action was observed with relation to the non-small lung cancer cell lines HOP-62 and HOP-92 with IC_50_ values of 0.3 and 1.7 µg/mL, respectively. Other types of cancer cell lines were also sensitive to compound **29** showing the following IC_50_ values: 2.4 and 3.6 µg/mL (colon 205 and HCT-116), 3.1, 3.9, and 5.4 µg/mL (renal RXF 393, 736-0 and ACHN); 4.1 µg/mL (leukemia HL-60(TB)); 5.9 µg/mL (breast HS 578T), 6.5 µg/mL (melanoma M-14); 6.8 µg/mL (CNS SF-539 and SNB-19); 7.1 µg/mL (ovarian OVCAR-8); and 8.4 µg/mL (prostate PC-3). 10-Substituted derivatives were less active in the preliminary test [73,83].

10*H*-2,7-diazaphenothiazine (**29**) and its nitrophenyl and pyrrolidynyloethyl derivatives exhibited a meaningful antioxidant activity (IC_50_ = 64–125 µM) in the experiment of non-enzymatic peroxidation of hepatic microsomal membrane lipids [84].

**Figure 16:**
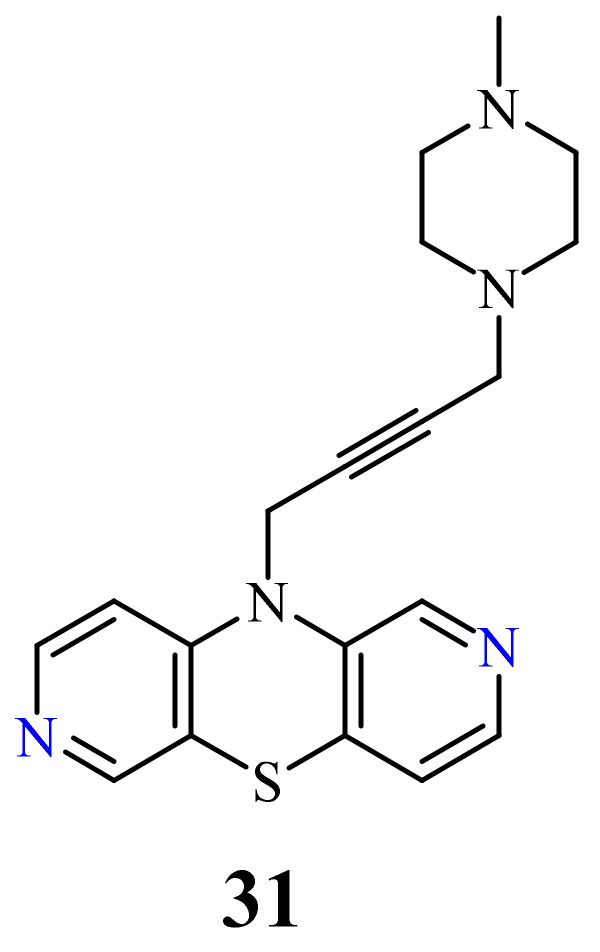
Anticancer active compound (**31**).

Another group of 2,7-diazaphenothiazine derivatives was obtained by a transformation of the 10-propargyl derivative of 2,7-diazaphenothiazine into the dialkylaminobutynyl derivatives. The anticancer activity was evaluated against the cell lines of human glioblastoma SNB-19, melanoma C-32, and breast cancer T-47D. The most active compound was the derivative **31** with the methylpiperazinylbutynyl substituent (Figure 16) against the carcinoma T-47D with the IC_50_ values of 9.6 µg/mL, being more potent than cisplatin (46.9 µg/mL). This derivative was also the most effective in relation to the glioblastoma SNB-19 (21.2 µg/mL). The remaining derivatives of this group showed a moderate activity with IC_50_ values in the range of 24.8 to 29.2 µg/mL. To understand the anticancer mechanism, the effect of derivative **31** on the expression of genes encoding *TP53, CDKN1A, BCL-2*, and *BAX* in the tested cancer cells was investigated. The RT-qPCR experiment showed the heightened number of *CDKN1A* copies in the T-47D and SNB-19 cells which implied the ability of the cell cycle arrest. Examination of the ratio of gene expression *BCL-2/BAX* revealed the activation of mitochondrial apoptosis in the T-47D cells [45].

**Figure 17:**
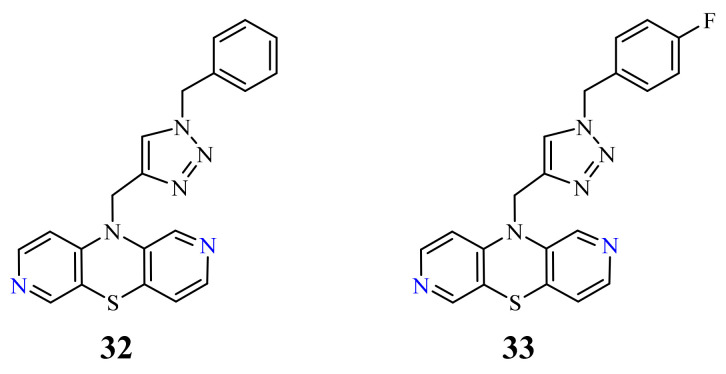
The most active hybrids of 2,7-diazaphenothiazines with the 1,2,3-triazole ring **32** and **33**.

The hybrids of 2,7-diazaphenothiazine and 1,2,3-triazole systems were obtained and examined for their cytotoxicity against the glioblastoma SNB-19, colorectal carcinoma Caco-2, lung cancer A549, and breast cancer MDA-MB231 cell lines (Figure 17). The anticancer activity of this group of derivatives was in the range IC_50_ = 0.26–49 μΜ. The most active compound was the derivative with benzyl substituent **32** in the 1,2,3-triazole ring showing the IC_50_ values of 0.26 μΜ against the Caco-2 and A549 cancer cell lines and 0.77 μΜ against the MDA-MB231 cancer cell line. A similarly high antiproliferative activity exhibited the derivative **33** with *p*-fluorobenzyl substituent 33 in 1,2,3-triazole ring against the MDA-MB231 (IC_50_ = 0.64 μΜ) and against the A549 (IC_50_ = 0.65 μΜ) cancer cell line [69].

Further studies looking for the structure–activity relationship (SAR) related to lipophilicity analysis were performed; however, they did not show the direct dependence of the anticancer activity on the lipophilic properties of 2,7-diazaphenothiazines [85].

### 3.5. 3,6-Diazaphenothiazines (Dipyrido[2,3-b;4’,3’-e][1,4]thiazines)

The 3,6-diazaphenothiazine system with the nitro group in position 1 and the chlorine atom and methoxy group in position 7 was described in the 1960s, but the identification of these compounds was based only on the logic of the conducted chemical syntheses. The author drew attention to the chemical properties of these molecules, while the biological activities were not described [86,87].

**Figure 18:**
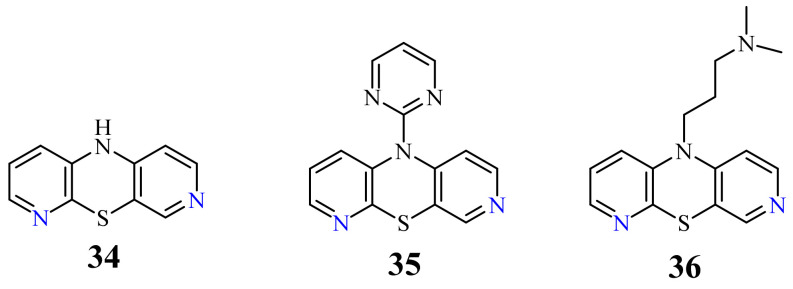
10*H*-,10-(2-pyrimidyl)- and 10-(dimethylaminopropyl)-3,6-diazaphenothiazines (**34**–**36**).

In 2016, a novel group of 3,6-diazaphenothiazines was described both due to interesting chemical and structural properties, but above all anticancer properties [46]. 10*H*-3,6-Diazaphenothiazine (**34**) (Figure 18) and its 10-derivatives containing varied substituents were tested for their anticancer activity. The parent compound **34** exhibited an extremely strong action against the glioblastoma SNB-19, melanoma C-32, and breast cancer MCF-7 cell lines with the IC_50_ values of 0.46 and 0.72 µg/mL. The derivative **35** (containing the 2-pyrimidinyl substituent, see Figure 18) showed a powerful activity against the breast cancer MCF-7 with the IC_50_ value of 0.73 µg/mL. Both derivatives were more effective than cisplatin. The derivative **36** (with the dimethylaminopropyl substituent) exhibited similar action to cisplatin (6.3 vs. 7.8 µg/mL) against the melanoma C-32 and moderate activity (11.3 µg/mL) against the breast cancer MCF-7. The family of these diazaphenothiazines showed non-toxic or almost non-toxic action against the normal fibroblast HFF-1 cell line [46].

The RT-qPCR experiment of gene expressions (*H3*, *TP53*, *CDKN1A*, *BCL-2*, and *BAX*) supported the antiproliferative action of diazaphenothiazines **34** and **35** and revealed the activation of the p53 pathway in cancer cells inducing the cell cycle arrest. Examination of the ratio of gene expression *BAX/BCL*-2 indicated the activation of mitochondrial apoptosis in the MCF-7 and SNB-19 cells [46].

Anticancer activity of 10*H*-3,6-diazaphenothiazine (**34**) was also studied on the A2780 ovarian cancer cells by an investigation on cytotoxicity profiles, the mechanism of apoptosis, and cell invasion. This parent compound induced a dose-dependent inhibition on the A2780 cancer cells (IC_50_ = 0.62 μM), with a significant less cytotoxicity towards the normal kidney HEK293 cells and the normal heart H9C2 cells. It was presented that the generation of reactive oxygen species (ROS) and the polarization of mitochondrial membrane potential (ΔΨm) suggested that compound **34** induced cell death through the oxidative damage. This compound elicited an upregulation of caspase-6, -3, and -7, which are actively involved in the formation of cell shrinkage, chromatin condensation, and the fragmentation of DNA. Additionally, the activation of caspase-3 brought about an increased enzymatic activity of DFFA (DNA fragmentation factor-α). This compound induced apoptosis via an intrinsic (mitochondria-dependent) and an extrinsic (cell death receptor-dependent) pathway. The inhibition of NF-κB and the subsequent inhibition of BIRC6-XIAP complexes reduced the invasion rate of the A2780 cancer cells penetrating through the MatrigelTM Invasion Chamber. The investigated compound exhibited a cytostatic action and significantly arrested cell proliferation at the G2/M phase. The presented results suggested that the 10*H*-3,6-diazaphenothiazines (**34**) would become promising chemotherapeutic agents in the future [74].

**Figure 19:**
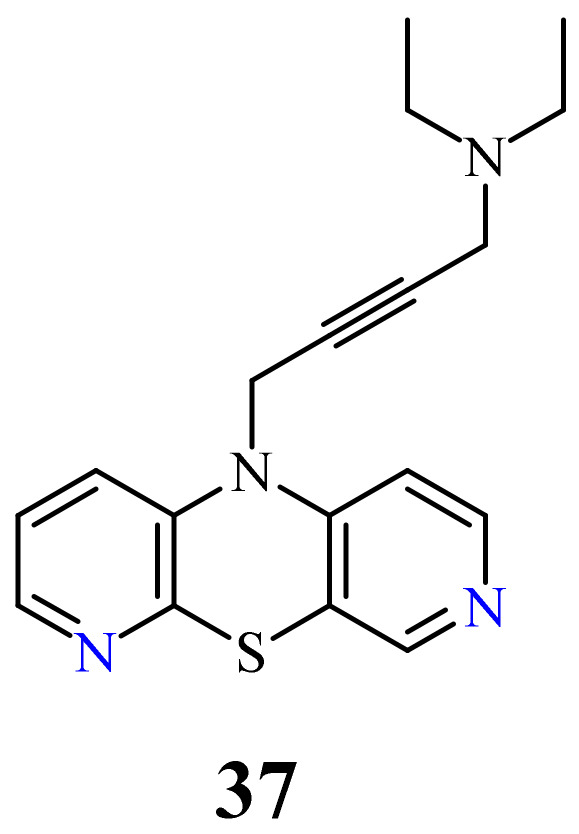
10-Diethylamino-2-butynyl-3,6-diazaphenothiazine (**37**).

Very recently, another group of new 10-substituted derivatives of 3,6-diazaphenothiazine, containing the triple bond linker ended with tertiary cyclic and acyclic amine groups was synthesized. This group exhibited varied anticancer activities against the human glioblastoma SNB-19, melanoma C-32, and breast cancer MDA-MB231 cancer lines, depending on the nature of the substituents. The most active compound was the derivative **37** with the diethylamino-2-butynyl substituent (Figure 19) against the glioblastoma SNB-19 (IC_50_ = 0.11 μg/mL). The analysis of genes expressions (*H3*, *TP53*, *CDKN1A*, *BCL-2*, and *BAX*) using the RT-qPCR method indicated the induction of mitochondrial apoptosis in the SNB-19 cells [75].

**Figure 20:**
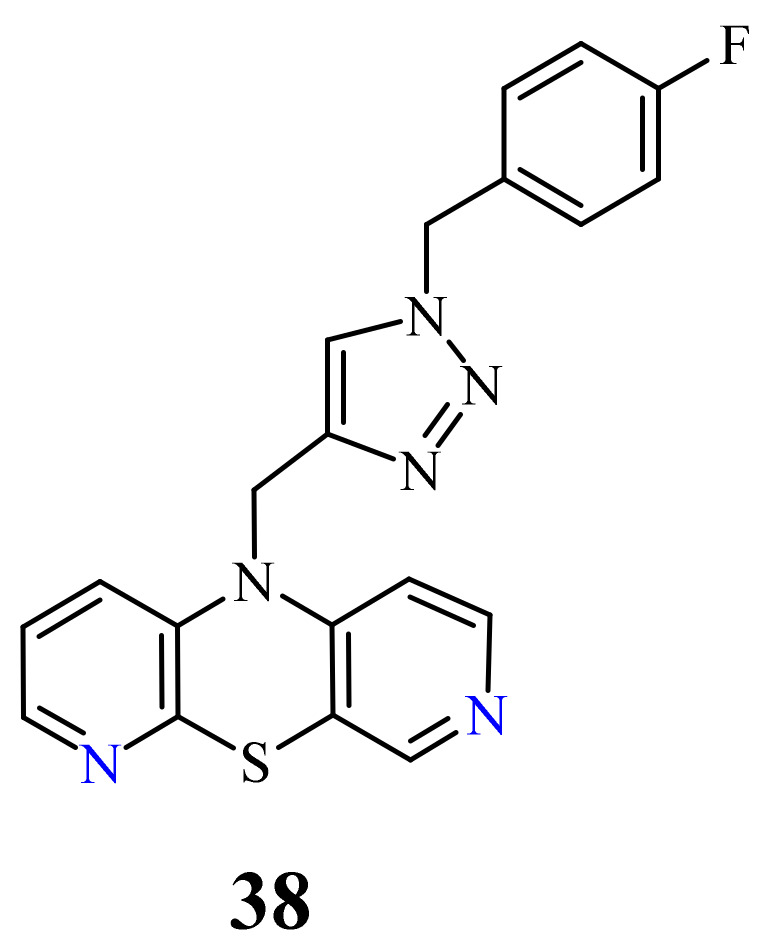
The most active hybrid of the 3,6-diazaphenothiazine and 1,2,3-triazole ring (**38**).

At the same time, novel 1,2,3-triazole-dipyridothiazine hybrids containing the 3,6-diazaphenothiazine system and selected *p*-substituted benzyl substituents at the 1,2,3-triazole ring were synthesized and screened for the anticancer activity. In this group of derivatives, the compound **38** with the *p*-fluorobenzyl substituent showed a very promising anticancer action (IC_50_ = 0.25 µM) in relation to the colorectal carcinoma Caco-2 and the lung cancer A549 (Figure 20). Additionally, the compound was nontoxic against the normal fibroblasts NHDF in the range of tested concentration [69].

In the group of 3,6-diazaphenothiazines, extensive studies of lipophilicity parameters and ADME properties were performed in order to look for the lipophilicity–activity relationship [83]. Nonetheless, a simple answer was not obtained as to which factor directly determines the high antitumor activity. The conducted research did not show a direct dependence of the anticancer activity on the lipophilic properties of this group of dipyridothiazines.

### 3.6. 3,7-Diazaphenothiazines (Dipyrido[3,4-b;4’,3’-e][1,4]thiazines)

**Figure 21:**
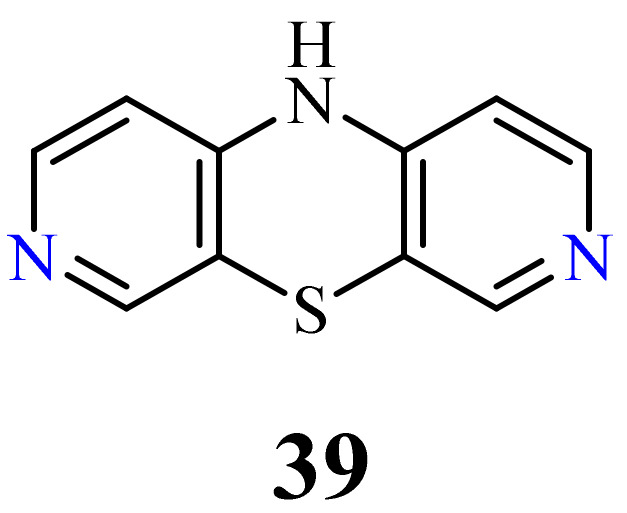
10*H*-3,7-diazaphenothiazines (**39**).

This group of diazaphenothiazines was described in the 1960s [88,89,90,91]. The parent compound 10*H*-3,7-diazaphenothiazine (**39**) and its 10-derivatives containing the diethylaminoethyl and dimethylaminopropyl substituents (Figure 21) exerted an antihistaminic action. Unfortunately, these activities were not substantially better than those of the leading phenothiazine drugs in the pharmaceutical market at the time. The anticancer activity of these compounds has not yet been evaluated.

Out of the six types of isomeric dipyridothiazines, five types were tested successfully against various kinds of cancer cell lines.

Most of the biological results discussed in this chapter were obtained by the authors in cooperation with international and domestic research groups.

The information provided on anticancer activity of pyridobenzothiazines and dipyridothiazines was summarized in Table 1.

## 4. Conclusions

The review summarizes the current knowledge on the anticancer activity of isomeric pyridobenzothiazines and dipyridothiazines, the modified azaphenothiazines with one and two pyridine rings against 10 types of cancer cell lines. Whereas pyridobenzothiazines were synthesized as modified successors of the classical neuroleptic phenothiazines and their anticancer activity was found only recently, dipyridothiazines were mostly obtained with the aim at the anticancer activity. The last type of compounds (of the isomeric 1,6-, 1,8-, 1,9-, 2,7-, and 3,6-diazaphenothiazine structures) exhibited very impressive activity with the IC_50_ values less than 1 µg/mL and 1 µM for some derivatives. Those compounds showed their anticancer action against various cancer lines of melanoma; leukemia; glioblastoma; and breast, colon, ovarian, renal, prostate, and lung cancers. The proteome profiling studies showed the most probable action through the intrinsic mitochondrial pathway of apoptosis, but in some cases also the extrinsic (cell death receptor-dependent) route is suggested. The structure–activity relationship of dipyridothiazines led to the conclusion that the strong anticancer action is dependent on both kinds of substituents at the thiazine nitrogen atom and the nature of the dipyridothiazine systems. These dipyridothiazine scaffolds are crucial for the anticancer activity as some N-unsubstituted dipyridothiazines were found to be very potent, for example, 10*H*-1,9-diazaphenothiazine and 10*H*-3,6-diazaphenothiazine. It also means this activity is relying on the location of the two azine nitrogen atoms in the tricyclic ring systems. The authors hope that this review highlights the importance of the pyridine modified phenothiazines in the search for lead compounds in the anticancer therapy.

## Figures and Tables

**Table 1:** Summarizing presentation of pyridobenzothiazines and dipyridothiazines and their anticancer activity.

Investigated Compounds	Substituents at the Thiazine Nitrogen Atom	Activity, IC_50_ [Ref]	Cancer Cell Lines
Pyridobenzothiazines			
1-Azaphenothiazine 3	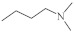	23.2 µg/mL [45,46,47] 28.1 µg/mL 32.3 µg/mL 36.6 µg/mL	C-32 MCF-7 T47D SNB-19
4	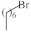	3.8–6.2 µg/mL [48]	H460,T98G, SNU80
6	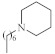	2.27–3.8 µg/mL [48]	H460, T98G, SNU80
7	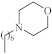	2.5 µg/mL [48]	H460
9	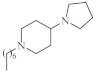	2.1–2.3 µg/mL [48]	H460, SNU80
Dipyridothiazines			
1,6-Diazaphenothiazine 13	H	4.8 µg/mL [47] 7.5 µg/mL	MCF-7 C-32
14	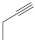	3.9 µg/mL [47]	MCF-7
15	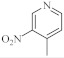	4.6 µg/mL [47]	MCF-7
16	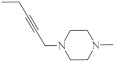	7.5 µg/mL [47]	MCF-7
17	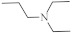	6.6 µg/mL [47]	C-32
18	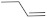	18.9 µg/mL [47]	SNB-19
19	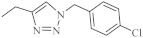	0.25-4.66 µM [69]	SNB-19, Caco-2, A549, MDA-MB231
1,8-Diazaphenothiazine			
20	H	5 µg/mL [70,71] 10 µg/mL	SW-948 L-1210.
23	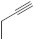	26-46 µg/mL [70]	SNB-19, C-32, T47D
24	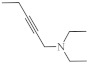	26.1 µg/mL [70]	C-32
25	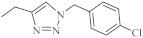	1.82 µM [69]	A549
1,9-Diazaphenothiazine			
26	H	3.83 µM [72]	C-32
27	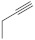	3.85 µM [72] 3.37 µM	SNB-19 C-32
28	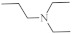	0.34 µM [72] 2.13 µM	SNB-19 MDA-MB-321
2,7-Diazaphenothiazine			
29	H	0.3 µg/mL [73] 1.7 µg/mL 2.4 µg/mL 3.6 µg/mL 3.1 µg/mL 3.9 µg/mL 5.5 µg/mL 4.1 µg/mL 5.9 µg/mL 6.5 µg/mL 6.8 µg/mL 7.1 µg/mL 8.4 µg/mL	HOP-62 HOP-92 colon 205 HTC-116 RXF 393 736-0 ACHN HL-60(TB) HS 578T M-14SF-539, SNB-19 OVCAR-8 PC-3
31	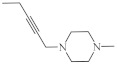	9.6 µg/mL [45]	T-47D
32	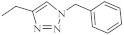	0.26 µM [69] 0.77 µM	Caco-2, A549 MDA-MB231
33	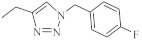	0.64 µM [69] 0.65 µM	MDA-MB231 A549
3,6-Diazaphenothiazine			
34	H	0.46 µg/mL [46] 0.72 µg/mL 0.62 µM [74]	SNB-19 C-32, MCF-7 A2780
35	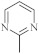	0.73 µg/mL [46]	MCF-7
36	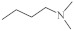	6.3 µg/mL [46] 11.3 µg/mL	C-32 MCF-7
37	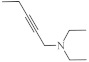	0.11 µg/mL [75]	SNB-19
38	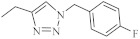	0.25 µM [69]	Caco-2, A549
